# Laws Regulating the Practice of Medicine

**Published:** 1874-05

**Authors:** 


WE HAVE RECEIVED the Constitution and By-Laws of the Association of the Alumni of the Albany Medical College. Alumni of the College are requested to forward the addresses of all graduates of the College within their knowledge to the Secretary of the Alumni Association, Dr. Willis G. Tucker, Albany, N. Y.

THE *Daily Commercial* makes its regular visits, from its home in the Mountain City, to our sanctum, sheds a genial glow over the breakfast table, lights up for the moment the grave editorial phiz, and beats lager-beer for appetite and good digestion. Its active "Head-Manager," and spicy, sharp-pointed "Local,"—deprived in his youth by sad accident of all but a *single understanding*—seems, in fortunate compensation, to be thereby enabled the better to concentrate himself upon *one point* and push forward the *Commercial* to an enviable success.

From the pennon editorial floats of late the name of Colonel S. Fouche, a name new to the *Commercial*, but old and war-worn in conflict of the political quill. Through a long life-time the Colonel has been widely known for his extraordinary skill in training succulent intellectual scions to a rigid and exact perpendicular. Indeed, we have repeatedly been assured by one of his former pupils, that the expressive countenance of the master was so interesting and fruitful a field of study, he never knew, during the five years of his pupilage, what boy sat behind him. If the Colonel could but wield the birch over the deeply-dyed political malefactors of the present day with his wonted zeal and rectitude of purpose and long accustomed success, a redeemed people would joyfully fill the air with anthems of enduring praise.

---

IN OUR GLEANINGS FROM EXCHANGES we present this month a poetical gem from the gifted pen of a distinguished barrister of this city, which we think worthy of being put into more permanent and enduring form than we now find it occupying in the columns of the Atlanta *Daily Herald*. We present it, too, as a specimen of very many excellent things which that enterprising and thrifty mirror of busy life brings every morning to our table. In this fast age, those who deny themselves the luxury of glancing over the newsy columns of the *Herald* at the breakfast table every morning, little know what they are really missing.

---

THE AMERICAN MEDICAL ASSOCIATION will hold its *twenty-fifth* annual session in Detroit, Mich., on Tuesday, June 2d, next, at 11 A. M. The permanent Secretary, Dr. Wm. B. Atkinson, No. 1400 Pine street, Philadelphia, Pa., requests that the Secretaries of all medical organizations that have adopted the Code of Ethics shall forward to him, as early as possible, a complete list of their officers, with their postoffice addresses, and the number of their members in good standing.

It will be seen by reference to the proceedings of the Georgia Medical Association at its recent meeting in Thomasville, that the subject of *laws regulating the practice of medicine* in the State was discussed in that body, and postponed for further action at its next annual meeting. The committee to which the matter was referred for consideration could agree upon but one point, and this a very important one, namely: the great importance of general unanimity of purpose and plan in the ranks of our profession, that we may have weight with the powers that be, and hope to secure wise and wholesome legislation upon the subject.

We know of no better means of arriving at the desired understanding of the important questions at issue, and of securing a happy concord of opinion and purpose among the members of the profession, than by the full and free expression of individual views and sentiments, and calm, friendly discussion of the whole subject through the columns of the medical press. Thus believing, we invite from our readers communications upon the subject for publication, and we hope that numerous responses will be made over the proper signatures of the writers. We trust that all selfishness will be laid aside, and that the questions involved will be viewed from the high stand points of the public weal and the honor and dignity of the profession, between which there is, and can be, no conflict. We shall take occasion to express our views of professional and public policy in this matter at an early day, and in the mean time desire to hear from our correspondents.

